# *Bulletin* comment: Learned helplessness^†^

**DOI:** 10.1192/pb.bp.114.047829

**Published:** 2014-08

Dogs repeatedly subjected to electric shocks sometimes give up trying to escape, and sit or lie quietly whining, waiting for the unpleasantness to stop. This description of how canines may react in certain circumstances was first made by psychologist Martin Seligman in his ground-breaking theory of learned helplessness which he believed could help explain the development of depression in humans.

Health professionals working in the current climate of continued cuts and savings are constantly being reminded of the gossamer-thin grip which they have on their jobs: endless meetings outlining the need to trim a little of this service or the freezing of another post in that department. Such a precarious position undoubtedly has an impact on how they might behave given the need to criticise the very institution or trust that employs them. To continue the dog theme, one is wary to bite the hand that feeds one.

Just over a year has passed since Robert Francis’s report on the tragic deficiencies at the Mid Staffordshire Trust was published and the issues of patient safety and quality of care have quite rightly taken centre stage in the headquarters of National Health Service organisations across the country. Improvements invariably cost: in real cash terms or lost opportunities. Such a situation puts even greater strain on an already stretched system.

In a response to the Francis report, the President of the Royal College of Psychiatrists warns clinicians they might be drifting into an insidious mindset of learned helplessness. She further states she believes that when health professionals do dare to put their heads above the parapet and speak out against cutbacks their voices are frail and inaudible.

To walk the fine line between ensuring high quality in the delivery of a service and making sure that management are not rubbed up the wrong way is, in the current climate, pretty hair-raising. To be criticised by leaders from whom support and guidance should be forthcoming is unhelpful. As any dog owner should know, the key to loyalty and respect is positive reinforcement.

